# DNA barcoding of Brazilian sea turtles (Testudines)

**DOI:** 10.1590/S1415-47572009005000050

**Published:** 2009-09-01

**Authors:** Sarah M. Vargas, Flávia C. F. Araújo, Fabrício R. Santos

**Affiliations:** Laboratório de Biodiversidade e Evolução Molecular, Instituto de Ciências Biológicas, Universidade Federal de Minas Gerais, Belo Horizonte, MGBrazil

**Keywords:** barcodes, sea turtles, *Cytochrome Oxidase c subunit I* (COI)

## Abstract

Five out of the seven recognized species of sea turtles (Testudines) occur on the Brazilian coast. The Barcode Initiative is an effort to undertake a molecular inventory of Earth biodiversity. *Cytochrome Oxidase c subunit I* (COI) molecular tags for sea turtle species have not yet been described. In this study, COI sequences for the five species of sea turtles that occur in Brazil were generated. These presented widely divergent haplotypes. All observed values were on the same range as those already described for other animal groups: the overall mean distance was 8.2%, the mean distance between families (Dermochelyidae and Cheloniidae) 11.7%, the mean intraspecific divergence 0.34%, and the mean distance within Cheloniidae 6.4%, this being 19-fold higher than the mean divergence observed within species. We obtained species-specific COI barcode tags that can be used for identifying each of the marine turtle species studied.

Marine turtles (Chelonioidea) are divided into two families, the mono-specific Dermochelyidae containing the leatherback turtle *Dermochelys coriacea*, and the Cheloniidae, with six recognized species divided into two tribes, Chelonini and Carettini. Chelonini is represented by the closely related green turtle *Chelonia mydas* and flatback turtle *Natator depressus*. Carettini includes the loggerhead turtle *Caretta caretta*, the olive and Kemp's ridley turtles, *Lepidochelys olivacea* and *L. kempi*, respectively, and the hawksbill turtle *Eretmochelys imbricata* (Ekert *et al.*, 1999; Plotkin, 2007).

The “Barcode of Life” Consortium is a worldwide initiative devoted to undertaking a molecular inventory of Earth biodiversity. After having been demonstrated that the Cytochrome Oxidase *c* subunit I (COI) gene of the mitochondrial DNA (mtDNA) could be successfully used for identifying North American bird species (Hebert *et al.*, 2004), many other vertebrate COI bar-codes have been produced (Vilaça *et al.*, 2006; Clare *et al.*, 2007; Chaves *et al.*, 2008).

Five out of the seven recognized marine turtle species occur on the Brazilian coast, namely the loggerhead (*Caretta caretta*), the hawksbill (*Eretmochelys imbricata*), the green (*Chelonia mydas*), the olive ridley (*Lepidochelys olivacea*) and the leatherback (*Dermochelys coriacea)* (Marcovaldi and Marcovaldi, 1999).

The large-scale sequencing of a single or few genes in taxonomic studies, denominated the Barcode initiative, aims at representing a practical method for species identification, as well as for providing insights into the evolutionary diversification of life (Stoeckle, 2003). Besides its usefulness in taxonomy, the barcode methodology is expected to be of great utility in conservation biology, for example, when performing biodiversity surveys. It could also be applied when traditional methods are inefficacious, as in the identification of eggs and larval forms, and in the analysis of stomach contents or excreta to determine food webs (Stoeckle, 2003). Furthermore, it can be potentially employed in forensic cases to identify the source of tissue samples obtained from the illegal commerce or use of eggs and meat.

Besides being eaten as food, in various human cultures the eggs of all turtle species are believed to possess aphrodisiac attributes. Turtle-egg consumption is believed to be the main cause for the severe decline in many sea turtle populations (Thorbjarnarson *et al.*, 2000). The use of turtle meat as food is also worthy of mention, especially in the case of green turtles which are widely hunted in such places as Costa Rica, Mexico, Venezuela, Australia and Papua New Guinea (Lefever, 1992; Kowarsky, 1995; Nabhan *et al.*, 1999). Thus, barcode methodology could be applied wherever turtle meat and eggs are eaten or trafficked, as a way of identifying species-source. In this way, barcode surveys could be used in sea turtle conservation and for alerting both the population and conservationists as to the local level of species-threat.

DNA barcodes could also be applied in field research for the identification of lost nests and turtles stranded on beaches and frequently encountered in an advanced state of decomposition, thereby complicating correct species identification. Another possibility is the rapid identification of interspecific hybrids, which can be as frequent as 45% in the population of *E.imbricata* of Bahia, Brazil (Lara-Ruiz *et al.*, 2006).

In this work, we generated COI barcodes and evaluated their usefulness for discriminating the five species of sea turtles occurring in Brazil.

Twenty six turtles from five species (*Lepidochelys olivacea* n = 3, *Chelonia mydas* n = 3, *Caretta caretta* n = 4, *Eretmochelys imbricata* n = 8 and *Dermochelys coriacea* n = 8) were sampled from beaches in different states of Brazil (Table 1). Several of the individuals had been used in prior studies on control-region sequences, namely from *E. imbricata* (n = 119) (Lara-Ruiz *et al.*, 2006), *D. coriacea* (n = 63) (Vargas *et al.*, 2008) and *C. caretta* (n = 84) (Reis *et al.*, unpublished), which thus favored our selection of the most divergent individuals within each species for the present analysis.

Fresh skin biopsies were taken from the front flipper of live animals and stored at room temperature in ethanol 70%, until total DNA was extracted as previously described (Lara-Ruiz *et al.*, 2006). For *L. olivacea*, the COI mitochondrial gene was amplified as a whole by using the specific primer SOCOF1 developed in our laboratory, as well as the H8121 primer designed by M. D. Sorenson (see Internet Resources). LCO1490 and HCO2198 primers developed by Folmer *et al.* (1994) were used for the remaining four species. PCR reaction mixes of 15 μL included 2 μL of genomic DNA (~ 40 ng), 1 U of *Taq* polymerase (Phoneutria^®^), 200 μM of dNTPs, 1X Tris-KCl buffer with 1.5 mM MgCl_2_ (Phoneutria^®^) and 0.5 μM of each primer. Before sequencing, the PCR products were cleaned by precipitation, using 20% polyethyleneglycol (Lara-Ruiz *et al.*, 2006). Sequencing reactions were carried out with each primer in a final volume of 10 μL containing 2 μL of purified PCR product, 3 μL of ultrapure water, 1 μL of primer (5 μM) and 4 μL of sequencing kit (ET DYE Terminator Kit, Amersham Biosciences). Sequencing products were precipitated with ammonium acetate and ethanol, dried at room temperature, dissolved with formamide-EDTA and run in a MegaBACE 1000 automatic sequencer (General Electric Healthcare).

Consensus sequences (# BBT001-08 to BBT026-08 are available in the BOLD database - see Internet Resources) were obtained and checked with the software Phred v. 0.20425 (Ewing *et al.*, 1998), Phrap v. 0.990319 and Consed 12.0 (Gordon *et al.*, 1998, see Internet Resources). Alignments were performed using Clustal X (Thompson *et al.*, 1997), with a manual edition, whenever necessary. Sequence divergence among different haplotypes was estimated with MEGA 3.0 software (Kumar *et al.*, 2004) by using the Kimura 2-parameter (K2p) distance model (Nei and Kumar, 2000). MEGA 3.0 was also used to construct a Neighbor-Joining (NJ) tree based on the K2p model with 10,000 bootstrap replicates.

A 589 bp COI fragment was analyzed in this study. The sequences obtained were aligned and compared with another GenBank COI sequence for *Chelonia mydas* (#AB012104). These comparisons revealed neither stop nor nonsense codons, neither were alignment gaps found. Although specimens of *D. coriacea* bearing six different control region haplotypes were analyzed, only one COI haplotype was encountered in this species. Different COI haplotypes were found for *E. imbricata* (four haplotypes) and *C. caretta* (three haplotypes), the average intra-specific K2P distances being 0.9% and 0.8%, respectively (Table 2), thereby exceeding values encountered in other studies, such as 0.6% for neotropical bats (Clare *et al.*, 2007) and 0.34% for Brazilian birds (Vilaça *et al.*, 2006). Nucleotide divergence among species was high, ranging from 6.3% for *L. olivacea* and *C. caretta* to 13.9% for *C. mydas* and *D. coriacea* (Table 2).

The overall mean distance was 8.2% and the mean distance between the families Dermochelyidae and Cheloniidae, 11.7%. The mean distance within Cheloniidae was 6.4%, 19 times higher than the mean divergence observed within species (0.34%). Only one haplotype was discriminated in Dermochelyidae, therefore the mean distance was zero. The COI gene sequences significantly discriminated all the species (99% bootstrap support), but did not split the two Cheloniidae tribes (Carettini and Chelonini), as can be seen in the NJ tree (Figure 1).

The autapomorphic sites discovered in each species are shown in Table 3. Most of the 71 autapomorphic sites were found in *D. coriacea* (32 sites), as expected, since Dermochelyidae diverged from the Cheloniidae in the late Cretaceous about 100 million years ago (Spotila, 2004). *E. imbricata* presented six autapomorphic sites, *L. olivacea* eight, *C. mydas* eighteen and *C. caretta* seven.

These autapomorphic characters, which are supposedly intraspecific synapomorphies, should be considered with caution since they were found in only a few individuals from each species, although we had pre-selected highly divergent mtDNA sequences. Some of these characters may thus not appear as synapomorphic in a broader sampling study.

The finding of characteristic species-specific COI sequences offers the prospect of identifying marine turtle species by using DNA barcode methodology as an auxiliary tool for taxonomy. This can also be used during field work, when identifying lost nests, animals stranded on beaches or those killed as part of the bycatch in fishery nets. A further use is in forensic litigation when turtle eggs or meat are the only available material.

**Figure 1:**
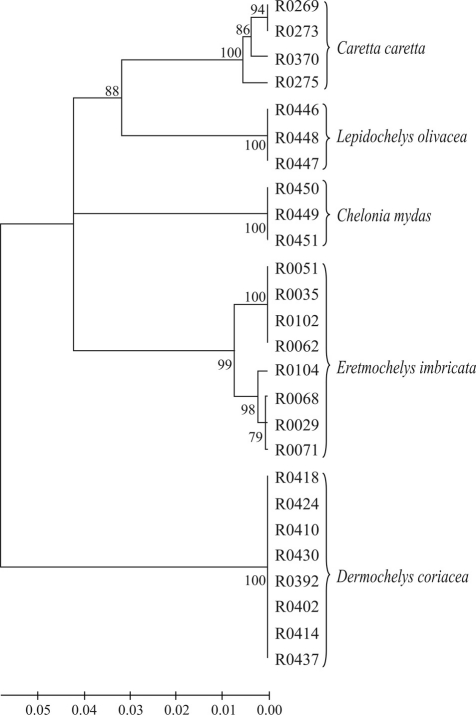
Neighbor-Joining tree of 589 COI sequences for five sea turtle species. Bootstrap support values are indicated on the branches. Three haplotypes were discriminated for *C. caretta* (n = 4), one for *Lepidochelys olivacea* (n = 3), one for *Chelonia mydas* (n = 3), four for *Eretmochelys imbricata* (n = 8) and one for *Dermochelys coriacea* (n = 8).

Despite these advantages, DNA barcoding must be employed with care in those sites where hybridization has already been registered, as in the hawksbill population of Bahia (Brazil), where 50 out of 119 individuals manifested two of the haplotypes characteristic of the loggerhead species (Lara-Ruiz *et al.*, 2006). Hybrid turtles display mitochondrial DNA characteristics of the maternal species against a background of the maternal and paternal nuclear genes from both species, thus composing a mosaic that may lead to incorrect taxonomic identification. Nevertheless, in most cases COI barcodes can be used for the trustworthy identification of maternal ancestry and testing the possibility of hybridization when discrepancies between morphology and expected mtDNA inheritance are encountered.

## Figures and Tables

**Table 1:** Sampling sites in Brazil and control region haplotypes.

Specie	ID	Location	Control region haplotype
*C. caretta*	R0269	Elevação do Rio Grande (Rio Grande do Sul)	A33*
*C. caretta*	R0273	Elevação do Rio Grande (Rio Grande do Sul)	A34*
*C. caretta*	R0370	Elevação do Rio Grande (Rio Grande do Sul)	A2*
*C. caretta*	R0275	Elevação do Rio Grande (Rio Grande do Sul)	A11*
*L. olivacea*	R0446	Praia Tigre da Base (Sergipe)	-
*L. olivacea*	R0448	Praia Ponta dos Mangues (Sergipe)	-
*L. olivacea*	R0447	Praia Tigre da Base (Sergipe)	-
*C. mydas*	R0450	Fernando de Noronha (Pernambuco)	-
*C. mydas*	R0449	Fernando de Noronha (Pernambuco)	-
*C. mydas*	R0449	Fernando de Noronha (Pernambuco)	-
*E. imbricata*	R0051	Praia do Forte (Bahia)	EiBR10**
*E. imbricata*	R0035	Atol das Rocas (Rio Grande do Norte)	EiBR12***
*E. imbricata*	R0102	Fernando de Noronha (Pernambuco)	EiBR16**
*E. imbricata*	R0062	Praia do Forte (Bahia)	EiBR9**
*E. imbricata*	R0104	Fernando de Noronha (Pernambuco)	EiBR14***
*E. imbricata*	R0068	Atol das Rocas (Rio Grande do Norte)	EiBR5***
*E. imbricata*	R0029	Atol das Rocas (Rio Grande do Norte)	EiBR6***
*E. imbricata*	R0071	Atol das Rocas (Rio Grande do Norte)	EiBR7***
*D. coriacea*	R0418	Elevação do Rio Grande (Rio Grande do Sul)	Dc_A2****
*D. coriacea*	R0424	Elevação do Rio Grande (Rio Grande do Sul)	Dc_I****
*D. coriacea*	R0410	Elevação do Rio Grande (Rio Grande do Sul)	Dc_A3****
*D. coriacea*	R0430	Elevação do Rio Grande (Rio Grande do Sul)	Dc_C****
*D. coriacea*	R0392	Elevação do Rio Grande (Rio Grande do Sul)	Dc_A1****
*D. coriacea*	R0402	Elevação do Rio Grande (Rio Grande do Sul)	Dc_A1****
*D. coriacea*	R0414	Elevação do Rio Grande (Rio Grande do Sul)	Dc_C****
*D. coriacea*	R0437	Elevação do Rio Grande (Rio Grande do Sul)	Dc_A4****

*Reis *et al.*, unpublished. **Lara-Ruiz *et al.*, 2006. ***Lara-Ruiz *et al.*, unpublished. ****Vargas *et al.*, 2008.

**Table 2:** Mean sequence divergence (K2P) within species (underlined number) and between pairs of sea turtle species found in Brazil.

	*Cm*	*Cc*	*Lo*	*Ei*	*Dc*
*Chelonia mydas (Cm)*	0.000				
*Caretta caretta (Cc)*	0.096	0.008			
*Lepidochelys olivacea (Lo)*	0.084	0.063	0.000		
*Eretmochelys imbricata (Ei)*	0.086	0.089	0.079	0.009	
*Dermochelys coriacea (Dc)*	0.139	0.122	0.124	0.103	0.000

**Table 3:** Autapomorphic characters (highlighted in black) found in all individuals within each sea turtle species.

Species	COI variable sites
	00000111111111111222222223333333333333444444444445555555555555555
	02568012345677889144558990011334566789000134446890011123344567888
	95401213910257698039895176926038409270258421478725867981703249258
*Dermochelys coriacea* (n = 8)	ACGCTCCCGTCCCTAAGACGTCTACCTTCACTCTTGTAATTCCTATATTTTATGATACCTCTTTT
*Chelonia mydas* (n = 3)	CTATCTT.A.TT.AT.ATTA..CTTA.CTGTC..CCATTCC..CCCCCC.C.CTCCCT..TCCC.
*Caretta caretta* (n = 4)	..AT....ACT.TA..ATTA..CC.GCCT...TCCCATT.C..CTCCC..CTCAC.TT.CTC...
*Lepidochelys olivacea* (n = 3)	..AT....A.A..AGGATTA.TCT.A.CT.....CCATC.CTTCTCCC.CCGCAT.TTT.TC...
*Eretmochelys imbricata* (n = 8)	..AT...TA....A..ATAAA.GC.A.CT..A..CCACC.C..CTCCC..C.CAT.CT..TC..C
